# Unveiling the Hidden Burden: From EPICOVIDEHA to EPIFLUEHA, Exploring the Epidemiology of Respiratory Viral Infections in Hematological Patients

**DOI:** 10.1097/HS9.0000000000000970

**Published:** 2023-10-19

**Authors:** Jon Salmanton-García, Francesco Marchesi, Federico Itri, Francesca Farina, Martin Hoenigl, Raúl Córdoba, Shaimma El-Ashwah, Alessandro Busca, Marianna Criscuolo, Oliver A. Cornely, Livio Pagano

**Affiliations:** 1 Faculty of Medicine and University Hospital Cologne, Institute of Translational Research, Cologne Excellence Cluster on Cellular Stress Responses in Aging-Associated Diseases, University of Cologne, Germany; 2 Department I of Internal Medicine, Faculty of Medicine and University Hospital Cologne, Excellence Center for Medical Mycology, University of Cologne, Germany; 3 German Centre for Infection Research (DZIF), Partner Site Bonn-Cologne, Germany; 4 Hematology and Stem Cell Transplant Unit, IRCCS Regina Elena National Cancer Institute, Rome, Italy; 5 San Luigi Gonzaga Hospital - Orbassano, Italy; 6 IRCCS Ospedale San Raffaele, Milan, Italy; 7 Division of Infectious Diseases, ECMM Excellence Center for Clinical Mycology, Department of Internal Medicine, Medical University of Graz, Austria; 8 BioTechMed, Graz, Austria; 9 Fundacion Jimenez Diaz University Hospital, Health Research Institute IIS-FJD, Madrid, Spain; 10 Oncology Center, Mansoura University, Mansoura, Egypt; 11 Stem Cell Transplant Center, AOU Citta’ della Salute e della Scienza, Turin, Italy; 12 Hematology Unit, Università Cattolica del Sacro Cuore, Rome, Italy; 13 University of Cologne, Faculty of Medicine and University Hospital Cologne, Clinical Trials Centre Cologne (ZKS Köln), Germany; 14 Faculty of Medicine and University Hospital Cologne, Center for Molecular Medicine Cologne, University of Cologne, Germany

Individuals with hematological malignancies face an ongoing risk of immunodeficiency, coming from both the malignancy itself and the therapies used to treat it. This increased vulnerability makes them susceptible to a range of pathogens, including bacteria,^1^ fungi,^2^ and viruses.^3^ Among these, respiratory viral infections have long been recognized as a significant contributor to morbidity and mortality in hematological patients,^4^ leading to severe respiratory complications, exacerbation of underlying hematological conditions, and potential compromise of treatment outcomes. Furthermore, respiratory viral infections often serve as the primary cause of hospitalization and even mortality within this patient population.^5–7^ Consequently, comprehending and effectively managing these infections are of paramount importance to optimize patient care and enhance outcomes.

Since its onset in December 2019, the coronavirus disease 2019 (COVID-19) pandemic,^8^ caused by severe acute respiratory syndrome coronavirus type 2 (SARS-CoV-2), has commanded the implementation of infection control measures, such as mask-wearing and social distancing, intended to curb COVID-19 transmission, may have contributed to a decrease in the overall occurrence of other respiratory viral infections.^9,10^ While COVID-19 shares similarities with other viral respiratory infections, it also exhibits distinct characteristics that set it apart. Like other respiratory viruses, SARS-CoV-2 is primarily transmitted through respiratory droplets and aerosols, causing similar symptoms such as fever, cough, and shortness of breath. However, SARS-CoV-2 stands out due to its higher transmissibility, severity, and potential for long-term complications such as post-acute sequelae of SARS-CoV-2 infection, the so-called long COVID. Simultaneously, it is essential to recognize the commonalities shared with other viral respiratory infections, such as the importance of early diagnosis, prompt treatment, and preventive measures to reduce the risk of infection in hematological malignancy patients.^11,12^ Understanding these similarities and differences is crucial for tailoring effective strategies to mitigate the impact of respiratory viral infections on this vulnerable population.

In response to the COVID-19 pandemic, the EPICOVIDEHA registry was established to collect epidemiological data on hematological malignancy patients infected with SARS-CoV-2.^13^ EPICOVIDEHA emerged as an initiative from the European Hematology Association Specialized Working Group Infections in Hematology in early 2020 with the aim to analyze and understand the epidemiology and outcome of hematological malignancy patients developing COVID-19. After 3 years, this collaborative effort with 314 contributors in 45 countries is evolving and will incorporate in its scope other community-acquired viral infections. By expanding its focus, EPICOVIDEHA will provide a comprehensive understanding of the impact of various respiratory viral infections on hematological patients. The expanded registry, named EPICOVIDEHA-EPIFLUEHA, will enable the assessment of the clinical characteristics and outcomes of patients with hematological malignancies affected by respiratory viral infections, such as those caused by influenza viruses, metapneumovirus, parainfluenza virus, respiratory syncytial virus, and rhinovirus. In parallel, EPICOVIDEHA-EPIFLUEHA will continue collecting details on SARS-CoV-2 infections. This will not only enhance our knowledge of these infections but also contribute to the formulation of evidence-based data for the management of the most common respiratory viral infections in hematological patients, allowing comparison between different viral infections.

EPICOVIDEHA-EPIFLUEHA will continue to employ specific inclusion and exclusion criteria to ensure the accuracy and focus of the research. To be included in the registry, patients must have had an active malignancy within the last 5 years before viral infection diagnosis. Furthermore, participants must be at least 18 years old to align with the current registry’s focus on adult patients. Additionally, laboratory-based diagnosis of SARS-CoV-2 infection will remain a requirement for inclusion in the registry. This criterion ensures that our research targets specifically on hematological patients who have confirmed cases of viral infection. Conversely, certain exclusion criteria currently in place will be maintained. Solid tumors will continue to be excluded from the study, so as non-malignant hematological diseases, and individuals with inactive or off-therapy malignancies within the last 5 years before the viral infection diagnosis.

This way, EPICOVIDEHA-EPIFLUEHA aspires to emulate the remarkable collaborative success achieved over the past 3 years by EPICOVIDEHA. The importance of acknowledging the contributions of patient participants will continue. As we move forward, authorships and collaboratorships will continue to be granted to contributors whose patients are included in the analysis and therefore are part of the findings and outcomes of EPICOVIDEHA-EPIFLUEHA. This collaborative effort not only enriches the research outcomes but also fosters a sense of ownership and engagement among all stakeholders. Moreover, contributors will continue having access to the complete patient sample, allowing them to pursue their own suggested research topics. This inclusive approach empowers researchers to explore their specific areas of interest within the EPICOVIDEHA-EPIFLUEHA framework (https://pubmed.ncbi.nlm.nih.gov/?term=epicovideha&sort=date&size=200).

Following in the footsteps of EPICOVIDEHA, EPICOVIDEHA-EPIFLUEHA aims to publish studies encompassing any facet of respiratory viral infections in hematological malignancy patients. EPICOVIDEHA publications targeted a range of topics, including comprehensive descriptions of the population affected, investigations into the impact of recent hematological treatments administered immediately before COVID-19 diagnosis, focused analyses for specific malignancy diagnoses, and exploration of treatment strategies for viral infections.

In summary, the expansion of the EPICOVIDEHA registry to the EPICOVIDEHA-EPIFLUEHA via the incorporation of any respiratory infection due to community-acquired respiratory viruses, comprehensive insights into the epidemiology and outcomes of these infections in hematological malignancy patients will be gained. This endeavor will inform evidence-based strategies to mitigate the risks associated with respiratory viral infections and improve patient care.

## ACKNOWLEDGMENTS

The authors express their deepest gratitude to all the contributing researchers and anonymous patients for their invaluable collaboration since 2020, which has hopefully promoted scientific progress and improved and saved countless lives. Their dedication, expertise, and selfless contributions have been instrumental in shaping the scientific landscape and fostering a culture of shared knowledge and progress.

## DISCLOSURES

The authors have no conflicts of interest to disclose.

## ENROLLMENT OF NEW CASES

EPICOVIDEHA-EPIFLUEHA welcomes new contributors. To take part in contributing new patients, kindly contact the corresponding author for further details or reach the registry through the website https://ehaweb.org/covid-19/epicovideha-survey/.

## SOURCES OF FUNDING

EPICOVIDEHA has received funds from Optics COMMIT (COVID-19 Unmet Medical Needs and Associated Research Extension) COVID-19 RFP program by GILEAD Science, United States (Project 2020-8223).
